# Editorial

**Published:** 1882-05

**Authors:** 


### Editorial.

In a recent editorial we were led to speak briefly upon the subject of the comparative interest shown by physicians here and abroad in the prosecution of original scientific studies. In accordance with our intention, at that time expressed, we now proceed to a discussion of the reasons for the disparity, at that time noticed. As we then remarked, the fault lies with the members of the profession and is to be found in their want of enthusiasm regarding scientific work. But why is it that the profession of this country lags in this respect? The reasons are numerous, and to a number of these the reader's attention is now called.

In the first rank we will place the national characteristics. The life and work of the student of nature is one of laborious, patient and persevering toil. The results are slow in coming and hardly earned, and the connecting and practical value of the discoveries are seen often only after long intervals of discouraging delay. Some of the difficulties attending work of this character were forcibly pointed out in the paper to which we alluded in the previous editorial. The American, characterized by his energy, enterprise and business tact, by his inclination for extensive undertakings, promising brilliant pecuniary results, is for these reasons not so well adapted for the work in question, as are certain other nationalities. On the contrary, the conditions required are enthusiasm in the acquirement of knowledge, a deep interest in scientific truths, a patience and perseverance that time does not influence and repeated discouragements but strengthens. We do not wonder, therefore, that in this department of usefulness the Germans, as a nation, outrank all others in the number and character of their workers. Our only hope then lies in our endeavors to modify these unfavorable national characteristics.

Whatever may incline to lessen our love for money or make us in a greater measure independent of pecuniary considerations, and whatever may increase our zeal in the cause of scientific progress, will tend to the removing of this serious obstacle to our favorable competition with other nations.

That the profession generally must act in this matter, is evident. Still we cannot help thinking that the greatest blame for the present unsatisfactory condition of things lies with those more particularly in power—the heads of our colleges. Therefore it is they more especially who are called upon to act.

That the medical profession of this country is already too numerous, and is yearly becoming more and more so, we firmly believe. Likewise we maintain that the acquirements of the average graduate justly provoke unfavorable criticism. Neither of these propositions admits of gainsaying as they are but too apparent to every unprejudiced observer; and all argument to the contrary but tends to establish them more clearly. Competition is the life of trade, and were the practice of medicine properly nothing more, perhaps the fuller the profession the more enterprising the doctors.

But is it not true that a too great rivalry, accompanied as it is with a care for one's own material advancement, and sometimes for existence even, incites to an unscrupulous method and fosters the development of the black arts in medicine? And is it not also undeniable that a constant and overweaning strife for pecuniary success, if not for bare existence, deadens the zeal and enthusiasm for scientific studies?

It is a well known fact that the interest in the study of nature increases in more than a direct ratio to the advance in acquired knowledge and improved insight into its truths. The higher the standard of medical education then, with which the graduates of our colleges start out, the greater the number who will continue in after-years to prosecute their search after truth.

To complete the discussion of this subject we may still again, at a future time, suggest the means which, in our opinion, will bring about this much to be desired result.

